# Isolation and Characterization of High-Ethanol-Tolerance Lactic Acid Bacteria from Australian Wine

**DOI:** 10.3390/foods11091231

**Published:** 2022-04-25

**Authors:** Gang Jin, Vladimir Jiranek, Aaron Mark Hayes, Paul R. Grbin

**Affiliations:** 1 School of Food and Wine, Ningxia University, Yinchuan 750021, China; 2 Engineering Research Center of Grape and Wine, Ministry of Education, Yinchuan 750021, China; 3 School of Agriculture, Food and Wine, The University of Adelaide, Waite Campus, Urrbrae, SA 5064, Australia; vladimir.jiranek@adelaide.edu.au (V.J.); aaronmarkhayes@hotmail.com (A.M.H.)

**Keywords:** malolactic fermentation, lactic acid bacteria, amplified fragment length polymorphism, ethanol tolerance

## Abstract

Lactic acid bacteria are very important in winemaking. In this study, 108 lactic acid bacteria isolates were obtained from high-ethanol-content (~17% (*v*/*v*)) Grenache wines during uninoculated malolactic fermentation (MLF). The 16S rRNA and species-specific PCR showed that 104 of these were *Oenococcus* *oeni*, three were *Lactobacillus hilgardii*, and one was *Staphylococcus pasteuri*. AFLP of *Hind*III and *Mse*I digests of the genomic DNA of the *O. oeni* strains was developed for the first time to discriminate the strains. The results showed that the method was a suitable technique for discriminating the *O. oeni* strains. Based on the cluster analysis, nine *O. oeni* strains were chosen for inclusion in an ethanol tolerance assay involving monitoring of optical density (ABS_600nm_) and viable plating. Several *O. oeni* strains (G63, G46, G71, G39) survived and grew well in MRS-AJ with 17% (*v*/*v*) ethanol, while the commercial *O. oeni* reference strain did not. Strain G63 could also survive and grow for 168 h after inoculation in MRS-AJ medium with 19% (*v*/*v*) ethanol. These results suggest that *O. oeni* G63, G46, G71, and G39 could potentially be used as MLF starters for high-ethanol-content wines. All three *L. hilgardii* strains could survive and grow in MRS-AJ with 19% (*v*/*v*) ethanol, perhaps also indicating their suitability as next-generation MLF starter cultures.

## 1. Introduction

Malolactic fermentation (MLF) is a critical biochemical process in winemaking. Generally occurring at the end of alcoholic fermentation, either inoculated with starter cultures of selected strains or undertaken by indigenous bacteria. During MLF, lactic acid bacteria (LAB) transform L-malic acid into L-lactic acid and carbon dioxide [1]. This biochemical conversion decreases wine acidity, improves quality, and increases the biological stability of wine [2]. While the growth of LAB in wine can have benefits, it can also be detrimental to wine quality. This depends on which species or strain grows and the stage of the winemaking process at which growth occurs [3]. *Oenococcus oeni* is regarded as the principal LAB responsible for MLF, but many studies showed that *Lactobacillus* spp. are typically present throughout in the grape and wine environment and during MLF [4,5]. du Toit et al. [6] report that some *Lactobacillus* spp. are suitable as a new generation of MLF starter cultures, with a number of strains commercially available.

Opportunities to identify further strains with enhanced capabilities exist because wine is a complex, harsh environment containing sugars (glucose, fructose, riboses), ethanol, organic acids, amino acids, fatty acids, phenolic compounds, sulfur dioxide (SO_2_), and low pH. Especially inhibitory to wine microorganisms is the combination of ethanol, low pH, and temperature along with SO_2_ [1,7,8]. In warm to hot climate wine regions, the potential alcohol content of the wine can already be high, but with issues related to climate warming, ethanol contents in wine have been increasing [9]. As a result, the incidence of ethanol inhibition of the initiation of MLF, slow progress, or even arrest of MLF is likely to increase [9,10]. Therefore, the selection of highly ethanol tolerant starter cultures for MLF is very desirable for the wine industry.

Reliable and efficient methods for strain identification and discrimination do exist [11]. Both 16S rRNA sequencing and species-specific PCR have been widely used to identify the LAB strains, but differentiation of strains of the same species can only be achieved by methods sensitive to minor genotypic differences. The amplified fragment length polymorphism (AFLP) technique, based on the detection of genomic restriction fragments, has proven to be the most useful tool so far for such strain discrimination in *O. oeni* and other LAB [11].

This study sought to isolate LAB from high-alcohol wine with a view to identifying ethanol tolerant strains that could be of benefit to winemakers. To achieve this, uninoculated MLFs were undertaken in Grenache wines (produced by either uninoculated or inoculated alcoholic fermentations (AFs)). In this way, a number of bacterial strains were isolated, and 16S rRNA and species-specific PCR analyses were used to identify LAB species. AFLP with the restriction enzymes *Hind*III and *Mse*I was used to genotype the *O. oeni* isolates. A selection of the isolated *O. oeni* strains and three *L. hilgardii* strains were characterized further in terms of their ethanol tolerance in laboratory processing experiments.

## 2. Materials and Methods

### 2.1. Isolation of Highly Ethanol Tolerant Bacteria from Wine

Four Grenache wines (Yalumba Wine Company, Angaston, Australia) undergoing an uninoculated MLF in 15 L sterilized stainless steel tanks were sampled every 48 h for the isolation of LAB. Briefly, 10 mL of each wine was serially diluted and plated onto MRS-AJ medium (i.e., MRS with 20% (*v*/*v*) apple juice) plus agar (2%)). Plates were anaerobically incubation at 30 °C for 7 days, and colonies were selected depending on their color, size, shape, edge, and smoothness of the surface; the selected colonies were re-streaked for purification, and a single colony was scraped off the plate and inoculated to fresh MRS-AJ. Then, the isolates were maintained with 1 volume of 80% sterile glycerol at −80 °C. Further details of the wines and bacteria isolated are reported in Table 1.

### 2.2. Bacterial DNA Extraction

Bacterial colonies were inoculated into 5 mL of MRS-AJ medium and incubated at 30 °C in a carbon dioxide incubator for 72 h. Bacterial cells were harvested by centrifugation (10,000× *g*, 5 min) from 5 mL of cultures, twice washed in 1 mL of TE (10 mM Tris–HCl pH 8.0, 1 mM EDTA), resuspended in 0.55 mL of TE containing 20 µL of 50 mg/mL lysozyme, and incubated at 37 °C for 1 h. RNAase (1.5 µL) was then added with a repeat incubation at 37 °C for 1 h, before 30 µL of 10% SDS and 15 µL proteinase K (14–22 mg/mL) were added followed by a further 3 h at 37 °C. Finally, 100 µL of 5 mol/L NaCl and 80 µL CTAB-NaCl were added, with incubation for 30 min at 65 °C. The crude DNA preparation was purified by performing 2 to 3 phenol/chloroform/isoamyl alcohol (25:24:1) and one chloroform/isoamyl alcohol (24:1) extraction. Genomic DNA was precipitated by adding two volumes of cold ethanol followed by washing with 70% (*v*/*v*) ethanol and air drying at room temperature. Finally, the DNA pellet was dissolved in TE buffer (60 µL).

### 2.3. Bacterial Species Identification

Bacterial isolates were preliminarily identified using standard methods, including cultural/cell morphology, Gram staining, and catalase reaction [12]. Universal primers 8F (5′-AGAGTTTGATCCTGGCTCAG-3′) and 1492R (5′-GGTTACCTTGTTACGACTT-3′) were used to amplify 16S rRNA. The reaction was performed in a final volume of 50 µL using the following amplification mixture: 10–100 ng DNA template, 1.5 µL DMSO, 10 µL 5× Hi-Fi buffer, 10 pmol of each primer, 1 mM dNTPs, and 1 U DNA polymerase (Velocity, Bioline). An initial 5 min denaturation at 98 °C was followed by 35 cycles of 30 s at 98 °C, 30 s at 58 °C, and 45 s at 72 °C, with a final extension of 10 min at 72 °C. Amplified products were analyzed by 1.0% (*v*/*v*) agarose gel electrophoresis. Before submission to the Australian Genome Research Facility (AGRF, Waite Campus) for sequencing, all PCR products were purified via Wizard plus SV minipreps Gel and PCR clean-up system (Promega).

Identification of *O. oeni* isolates at the species level was performed by PCR with species-specific primers On1 (5′-TAATGTGGTTCTTGAGGAGAAAAT-3′) and On2 (5′-ATCATCGTCAAACAAGAGGCCTT-3′) according to Zapparoli et al. [13], which allowed amplification of the *mle* gene (1025 bp). PCR products were resolved by electrophoresis in 1.0% (*v*/*v*) agarose gels. Species-specific primers H2 (5′-ACTNATTTGACATTAAGA-3′) and 8623 (5′-CTGGTTCACTATCGGTCT- C-3′) were used to identify *L. hilgardii* [14]. PCR reactions in a total volume of 50 µL contained 10–100 ng DNA template, 1.5 µL DMSO, 10 µL 5× Hi-Fi buffer, 5 pmol of each primer, 0.8 mM dNTPs, and 1 U DNA polymerase (Velocity, Bioline). Reactions involved an initial 95 °C for 5 min followed by 30 cycles of 95 °C for 30 s, 36 °C for 30 s, and 72 °C for 2 min, and a final extension of 72 °C for 5 min. PCR products were resolved by agarose gel electrophoresis.

### 2.4. Amplified Fragment Length Polymorphism (AFLP) Analysis of *O. oeni*

Approximately 200 ng of DNA in a final volume of 20 µL was digested with 5 U *Mse*I and 10 U *Hind*III restriction enzyme (New England Biolabs, Ipswich, MA, USA) at 37 °C for 4 h. Digested DNA (20 µL) was transferred to a new tube containing 5 pmol of each *Mse*I adapter (5′-GACGATGAGTCCTGAG-3′; 5′-TACTCAGGACTCAT-3′), 2.5 pmol of each *Hind*III adapter (5′-CTCGTAGACTGCGTACC-3′; 5′-AGCTGGTACGCAGTC-3′), and 10 U DNA T4 Ligase (New England Biolabs) in a final volume of 30 µL and incubated overnight at 16 °C. A pre-selective PCR (94 °C 2 min, 30 cycles of 94 °C 30 sec, 56 °C 60 sec, 72 °C 1 min, and a final extension of 72 °C for 7 min) was carried out in a 25 µL (final volume) mixture contained 4 µL of DNA ligation (1: 5 dilution), 2.5 mM MgCl_2_, 0.2 mM dNTPs, 0.4 µM of each primer (H0: 5′-GACTGCGTACCAGCTT- 3′; M0: 5′-GATGAGTCCTGAGTAA- 3′), and 1.5 U DNA polymerase (Mango, Bioline, Eveleigh NSW, Australia). For the selective PCR reaction, a 1:20 dilution of digested–ligated DNA (2 μL) was amplified in a 25 μL (final volume) mixture using selective primers. Eleven separate primer combinations were used for the selective amplification to choose the best primer combination (Table 2). The PCR cycling conditions for these reactions were 94 °C 2 min, 10 cycles of 66 °C 30 s (down 1 °C each cycle) and 72 °C 2 min, followed by an extra 20 cycles at 94 °C 20 s, 56 °C 20 s, 72 °C 2 min, and a final extension at 60 °C for 30 min. PCR products were diluted 30 times before submission to the AGRF to carry out fragment analysis by capillary electrophoresis. Gene Mapper software version 3.7 (PE Applied Biosystems, Waltham, MA, USA) was used to automatically size and quantify individual fragments by using the internal lane standard. Peak height thresholds were set at 200. Genotyper software (PE Applied Biosystems) was set to heavy smoothing. Bands of the same size in different individuals were assumed to represent the same allele. Bands of different sizes were treated as independent loci with two alleles (present or absent). Data were reported in a binary format (1 = presence of a band/peak; 0 = absence). For clustering, fragments between 50 and 500 bp were analyzed with NTSYS software by using the Dice similarity coefficient based on presence/absence of the bands and clustered by the unweighted pair group method with arithmetic mean (UPGMA) [15].

### 2.5. Growth Assay in MRS-AJ

As a reference, approximately 10^7^ cfu of each strain was inoculated into 10 mL of MRS-AJ medium (pH 6.5). A 200 µL sample was taken at 0, 12, 18, 25, 35, 42, 48, 60, and 72 h of incubation at 28 °C. Culture development was estimated (Abs_600_), and the data were analyzed using GraphPad Prism 6.

### 2.6. Ethanol Tolerance Assay

Based on the AFLP analysis (i.e., distinctness of strains) and their robust growth properties, *O. oeni* strains G24, G39, G46, G52, G61, G62, G63, G71, and G107 and the three *L. hilgardii* strains G76, G102, and G103 were selected for the ethanol tolerance assay. *Oenococcus oeni* strain VP41 (Lallemand Inc., Montréal, QC, Canada) was used as the control strain due to its reported high tolerance to ethanol (Lallemand web site).

Approximately 10^7^ cfu of each strain was inoculated in triplicate into 50 mL MRS-AJ medium (pH 6.5) with 15%, 17%, and 19% (*v*/*v*) ethanol. Samples (200 µL) were taken every 24 h for 15% (*v*/*v*) ethanol ferments. For 17% and 19% (*v*/*v*) ethanol ferments, samples were taken at 24 and 48 h and then every 48 h. The Abs_600_ was determined, and data were analyzed using GraphPad Prism 6. A lack of detectable growth (Abs_600_ ≤ starting Abs) after 7 days was designated a non-culturable concentration.

As Abs_600_ measurements do not easily detect low cell numbers [16], plating on to MRS-AJ medium was performed to more accurately determine the extent to which ethanol concentration influenced the culturability of the selected bacterial isolates. Approximately 10^7^ cfu of each strain was inoculated in triplicate into 50 mL MRS-AJ medium (pH 6.5) with 15%, 17%, and 19% (*v*/*v*) ethanol. Samples (20 µL) were taken at 24, 110, 168, 210, and 309 h, serially diluted in 180 µL MRS medium (10^−1^~10^−5^) in a 96-well plate, and 5 µL of each dilution was drop inoculated onto solidified MRS-AJ medium and incubated anaerobically at 30 °C for 7 days. The resulting colonies were counted, and data were analyzed by GraphPad Prism 6.

### 2.7. Statistical Analysis

Analyses of variance were performed using StatGraphics Plus version 4.0 (Manugistics, Rockville, MD, USA). Significant differences were determined using Duncan’s test at *p* < 0.05.

## 3. Results

### 3.1. Isolation and Characterization of High Ethanol Tolerant Strains

A total of 108 isolates were recovered from four individual Grenache wines (~17% (*v*/*v*) ethanol) during uninoculated MLF. All strains were tentatively identified as lactic acid bacteria (LAB) on the basis of Gram stain positivity, catalase negativity, and cultural and cellular morphology. Identification of LAB isolates to the species level was performed by 16S rRNA sequence analysis and species-specific PCR. The data revealed that 104 strains were *O. oeni*, 3 were *L. hilgardii*, and 1 was *S. pasteuri* (Table 2).

### 3.2. Genotyping of *O. oeni* Strains by AFLP Analysis

The amplification efficiency obtained by the *Hind*III and *Mse*I digestion was higher than that with *EcoR*I and *Mse*I reported in the literature [17]. Characterization of the 104 *O. oeni* and 3 *L. hilgardii* isolates was carried out by AFLP analysis using *Hind*III– and *Mse*I–digested genomic DNA. Each primer combination produced an average of 61.6 amplification products per strain (85 with MA-HT FAM and MT-HT FAM to 34 with HA-MT HEX). Most primer combinations produced a high number of amplifications, with the primer pair MA-HT FAM and MT-HT FAM producing the most selective primer combinations (70–85), whereas the primer pair HA-MT HEX proved to be less selective (34–49) (Table 3).

For testing the reproducibility of the primer pair MA-HT and MT-HT, three *O. oeni* strains and three *L. hilgardii* strains were selected to repeat AFLP four times. The results showed that each primer pair tested had a reproducibility higher than 99% for *O. oeni* strains, while it was lower than 97% for *L. hilgardii* strains (Table 4).

**Figure 1:**
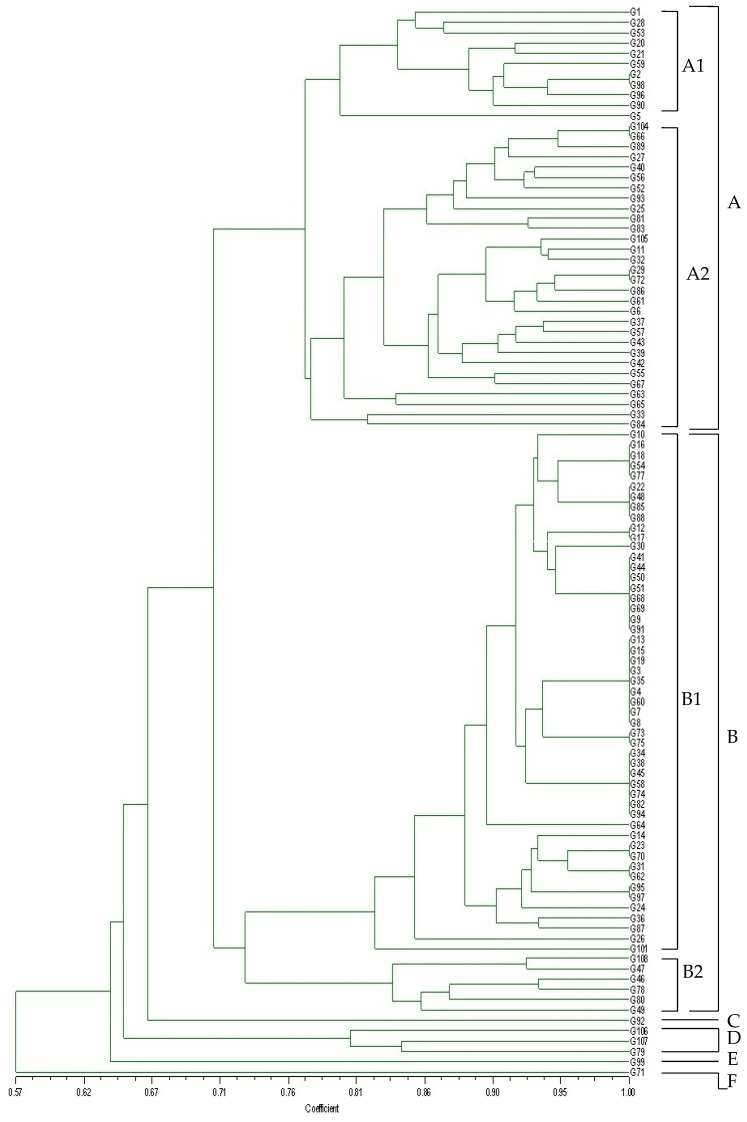
UPGMA dendrogram derived from the combined AFLP patterns using two different primer combinations of 104 *O. oeni* strains isolated from Australian Grenache wines. (six different principal clusters (A–F) were divided at the genetic similarity level of 71%).

UPGMA cluster analysis and a dendrogram were produced from the data obtained by the AFLP assay of 104 *O. oeni* isolates with the primer pair MA-HT FAM and MT-HT FAM. The genetic distance between each genotype (Figure 1) allowed the strains to be divided into six different principal clusters (A–F) at the similarity level of 71%. Two main clusters (A and B) included most of the *O. oeni* strains. Two major biotypes (A1 and A2) comprised 11 and 31 strains, respectively. Cluster A had variable AFLP similarity among the 42 isolates grouped therein (70%). Biotype A1 revealed an AFLP similarity ranging from 80% to 93%, whereas the similarity level of biotype A2 ranged from 77% to 95%. Cluster B also include two biotypes (B1 and B2), with biotype B1 being comprised of the most isolates (51 strains) and showing a higher AFLP similarity (82% to 95%); whereas the similarity level of biotype B2 ranged from 83% to 93%. G92 was the only strain in Cluster C, which gave an AFLP similarity of 66%. Cluster D included three strains that showed an AFLP similarity of 65%, making it the most variable. Cluster E comprised only one strain (G99) with an AFLP similarity of 64%. G71 showed the lowest AFLP similarity (57%) with other clusters. Therefore, AFLP with *Hind*III and *Mse*I was successfully able to reproducibly genotype *O. oeni*.

### 3.3. Ethanol Tolerance Analysis

**Figure 2:**
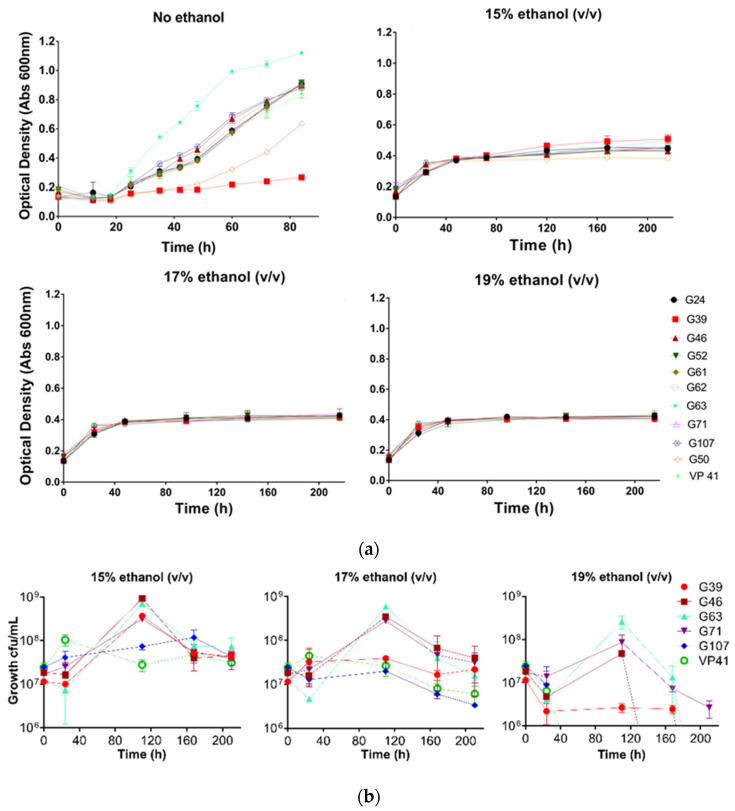
Growth of *O. oeni* isolates in MRS-AJ that contained 15%, 17%, and 19% (*v*/*v*) ethanol, respectively, determined by (**a**) Abs_600_; (**b**) drop plate counting (bars represent one standard deviation (*n* = 3)).

**Figure 3:**
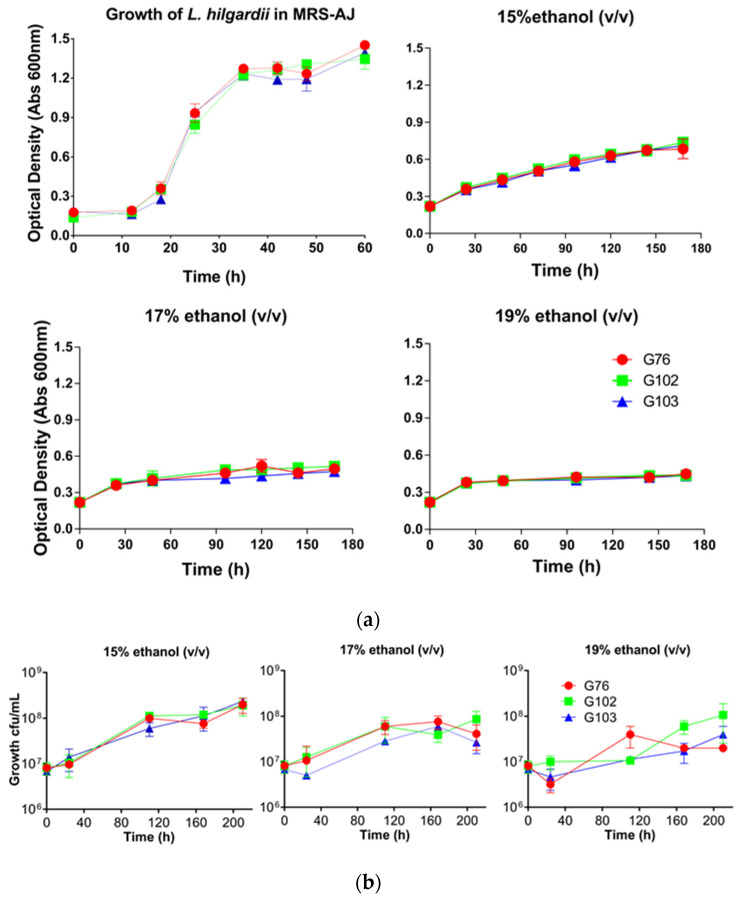
Growth of *L. hilgardii* isolates in MRS-AJ that contained 15%, 17%, and 19% ethanol respectively, determined by (**a**) Abs_600_; (**b**) drop plate counting (bars represent one standard deviation (*n* = 3)).

The growth of the *O. oeni* strains in comparison with a commercial reference strain (VP 41) was tested in growth studies in MRS-AJ (Figure 2a). Generally, the strains showed a steady growth that had not attained stationary phase after 80 h. *O. oeni* G63 had the fastest growth rate, and *O. oeni* G39 the slowest. After an initial lag of ~40 h, G50 appeared to grow at a similar rate to VP 41 and the remainder of the tested strains. The three *L. hilgardii* strains grew in a similar manner to one another, with a ~15 h lag followed by growth before a stationary phase commencing at ~35 h (Figure 3a).

The growth of various *O. oeni* (Figure 2a) and *L. hilgardii* strains (Figure 3a) inoculated into MRS-AJ medium containing 0%, 15%, 17%, or 19% (*v*/*v*) ethanol was characterized by Abs_600_. Growth was apparent for all strains. Inclusion of ethanol dramatically reduced growth and maximum optical density, with greater inhibition apparent at higher ethanol contents. The greatest resolution of the strains in the presence of ethanol was possible at 15% (*v*/*v*) ethanol, wherein the strains *O. oeni* G39 and *O. oeni* G63 had the fastest growth rate, surpassing the control strain *O. oeni* VP41 (Figure 2a). *O. oeni* G50 was the slowest growing isolate. The three *L. hilgardii* strains had a similar growth rate to one another in MRS-AJ medium with 15% (*v*/*v*) ethanol, and the exponential phase ended at about 170 h (Figure 3a). All *O. oeni* and *L. hilgardii* strains could grow in the 17% (*v*/*v*) ethanol MRS-AJ medium, albeit modestly. While all *O. oeni* strains could survive and grow in the 19% (*v*/*v*) ethanol MRS-AJ medium, there was little difference among these strains before 96 h (Figure 2a). All three *L. hilgardii* strains grew well in 19% (*v*/*v*) ethanol MRS-AJ medium; however, maximum Abs_600_ was not reached for more than 400 h (Figure S1, Supplementary Materials), compared with the 15% and 17% (*v*/*v*) ethanol treatment, where maximum Abs_600_ occurred after approximately 170 h (Figure 3a).

The viable plating results of *O. oeni* (Figure 2b) and *L. hilgardii* (Figure 3b) strains growing in MRS-AJ containing various ethanol concentrations indicated that *L. hilgardii* strains and some of the *O. oeni* strains had good tolerance to high ethanol. All *O. oeni* strains could grow in 15% (*v*/*v*) ethanol MRS-AJ medium, with the culturable cell numbers for all strains being higher than at inoculation after 210 h (Figure 3b). The same was true after this time for the 17% (*v*/*v*) ethanol MRS-AJ medium for *O. oeni* G63, G46, G71, and G39. However, the control strain *O. oeni* VP41 did not grow well, decreasing in culturable cell numbers from ~120 h. *O. oeni* G63, G46, and G71 could survive and grow in 19% (*v*/*v*) ethanol MRS-AJ medium until 110 h, but 58 h later, only *O. oeni* G63 was still culturable. By 210 h, no culturable *O. oeni* strains were detected. *O. oeni* VP41 was unable to survive these conditions. All three *L. hilgardii* strains grew in 15%, 17%, and 19% (*v*/*v*) ethanol MRS-AJ medium; however, higher culturable cell numbers were obtained in the 15% (*v*/*v*) ethanol treatment.

*O. oeni* strains G63, G46, G71, and G39 all displayed strong ethanol resistance along with the *L. hilgardii* strains. The *L. hilgardii* strains also grew in MRS-AJ medium that contained 19% (*v*/*v*) ethanol.

## 4. Discussion

In this study, the isolation and characterization of lactic acid bacteria from uninoculated malolactic fermentation of Grenache wines that had high ethanol content was carried out. In all, 104 *O. oeni*, 3 *L. hilgardii*, and 1 *S. pasteuri* strains were isolated from these wines. For the first time, a highly reproducible AFLP method using restriction enzyme *Hind*III and *Mse*I was developed for typing within *O. oeni* species. The ethanol tolerance of *O. oeni* and *L. hilgardii* strains was tested to highlight strains with potentially high ethanol tolerance.

*O. oeni* is one of the most representative species of LAB found in must and wine, and generally, its population increases during the fermentation [1]. Isolation and screening of superior *O. oeni* strains from grape and wine environments is still the most important way to make MLF starters. Lòpez et al. [18] identified *O. oeni* in 201 out of 204 isolates from wine samples. Cappello et al. [17] recognized 87 *O. oeni* strains out of 220 LAB from Primitivo wine during uninoculated MLF. The use of dependable and efficient methods for the identification and discrimination of bacterial strains is essential. Importantly, *O. oeni* strain differentiation can only be achieved by techniques that are sensitive to minor genotypic differences. A range of molecular biology methods have been utilized for *O. oeni* strain differentiation. Pulsed-field gel electrophoresis (PFGE) has excellent discriminative power for *O. oeni* strains [19,20,21], but this method is labor intensive and technically demanding, requiring specific apparatus, thereby likely restricting it to research and reference laboratories [22,23]. Compared to PFGE, random-amplification polymorphism DNA (RAPD) is quick and relatively easily performed. However, RAPD has several drawbacks as an amplification technique, such as reproducibility between laboratories [24,25].

AFLP has shown greatest usefulness so far for intraspecies discrimination of bacteria, including LAB [23,26,27]. Until now, most AFLP research on *O. oeni* used *EcoR*I and *Mse*I; however, the greater frequency of the adenine (A) + thymine (T)–rich recognition sequences of these enzymes in cytosine (C) + guanine (G)–poor genomes, reduces their resolving power. To address this, we modified and validated a rapid and simple method to identify *O. oeni* [11,13] and *L. hilgardii* [14] at a species level using *Hind*III instead of *EcoR*I. *Hind*III appeared more suited to digest genomic DNA with a G + C content of 40–50 mol%, while digestion with the ‘frequent-cutter’ *Mse*I was optimal for G + C contents below 50 mol% [28]. *Hind*III and *Mse*I were considered a good combination for *O. oeni* given a G + C content of around 40%. The results confirmed that the amplification efficiency was higher than the *EcoR*I and *Mse*I combination from the literature [17], with very good repeatability. Therefore, we conclude that AFLP with *Hind*III and *Mse*I is a useful technique to discriminate *O. oeni* strains. Notably, the repeatability of *L. hilgardii* was poorer (around 85%).

Due to the fact that high ethanol content often causes sluggish or stuck MLF in wine, the selection of highly ethanol tolerant strains is of interest to wine producers in hot-climate regions to help avoid such fermentation issues. Normally, *O. oeni* grows poorly when the ethanol exceeds 14% (*v*/*v*) [29]. In this study, all isolated strains investigated grew well in MRS-AJ containing 15% (*v*/*v*) ethanol. Some strains (G63, G46, G71, G39) could survive and grow for at least 210 h when inoculated into media containing 17% (*v*/*v*) ethanol, with G63 producing the highest quantity of culturable cells. G63 survived and grew for up to 168 h after inoculation into MRS-AJ containing 19% (*v*/*v*) ethanol. *O. oeni* isolates G63, G46, G71, and G39 outperformed the high-ethanol-tolerant commercial strain tested (*O. oeni* VP41, prepared as a fresh culture). More work is needed to determine whether these strains are also tolerant of other stresses (e.g., low pH, high SO_2_, low temperature), produce harmful metabolites, or have potential as MLF starter cultures under industrial conditions in high-ethanol wine fermentations.

Several wine LAB genera other than *Oenococcus*, including *Leuconostoc*, *Lactobacillus,* and *Pediococcus*, also decarboxylate L-malic acid into L-lactic acid and CO_2_. Emerging research is revealing that *Lactobacillus* spp. can be important in winemaking. Izquierdo et al. [4] and Ruiz et al. [5] found that non-*Oenococcus* species present during MLF consisted of *L. casei*, *L. hilgardii*, *L. plantarum,* and *Leuc. mesenteroides*. The enzyme responsible for malolactic fermentation, encoded by *mle*A, differs between the lactobacilli and the oenococci [30]. This may bring about differences in the acidity, microbial stability, and sensory complexity of wines produced using these and, thus, potentially influence wine quality.

A recent patent [31] relates to *Lactobacillus* and *Pediococcus* capable of completing MLF of wines at 10% (*v*/*v*) ethanol or more and high pHs, upon direct inoculation to 10^6^–5×10^7^ cfu/mL, whether in a dried, frozen, or lyophilized state. These strains had been selected for tolerance to various limiting conditions, especially for high ethanol. *L. plantarum* strain V22 assessed over three vintages at high pH (>3.5) and ethanol (≥14% *v*/*v*) [32] was also as fast as an *O. oeni* starter when inoculated post-AF. Such strains are of interest as they may possess more diversity in enzymatic activities (e.g., ochratoxin A reduction, β-glucosidase, esterase, protease, phenolic acid decarboxylase) compared to *O. oeni* [30]. Closer to the work reported here, *L. hilgardii* and *L. plantarum* were able to complete MLF (co-inoculated or post-AF) in wines at pH ~3.3, 13.5–16.5% (*v*/*v*) ethanol, and 1.2–3.2 g/L malic acid at 20 °C, at a rate equivalent to a commercial *O. oeni* starter culture [6]. Importantly, volatile aroma assays by an informal tasting panel revealed that these *Lactobacillus* strains produced different profiles to *O. oeni*, with no off-flavors detected [33]. Others showed that *L. plantarum* improved wine color during MLF [34]. Accordingly, such lactobacilli offer promise as new starters and may be more suitable for high-pH and high-ethanol wines.

As part of this study, three *L. hilgardii* strains were isolated as non-*Oenococcus* species present in high-pH (>3.5) and high-ethanol wines during uninoculated MLF. The ethanol tolerance assay (Figure 3) determined that they could grow well in MRS-AJ that contained 19% (*v*/*v*) ethanol, which was much higher than previously reported [35]. Nowadays, climate warming can mean greater grape maturity at harvest resulting in wines with higher pH and ethanol, which have a critical impact on the MLF of wines [9,36]. Therefore, it is of great importance to further understand whether these *L. hilgardii* strains have potential as MLF starter cultures in future wines, which may have stressful pH values and ethanol contents.

Interestingly, an *S. pasteuri* strain was isolated from the AU4 barrel (Table 1). There are only a few reports regarding *S. pasteuri;* therefore, it has not been clearly described. *S. pasteuri* is a coagulase-negative staphylococci, which has been isolated previously from naturally fermented Italian sausages, in which its activity impacts color and aroma development, pathogen inhibition, and shelf-life enhancement [37]. *S. pasteuri* is one of the few microorganisms cultured from the stratosphere [38], it is not a common skin contaminant [39,40], and has never been found responsible for human disease. Although it was found as a joint-prosthesis-colonizing organism, its pathogenic role as a cause of arthroplastic infections remains unclear [41]. Jisoo et al. [42] found that *S. pasteuri* produces one or more new antimicrobial substances, which are putative new bacteriocins and candidates for use in the control of food-borne pathogens, such as antibiotic-resistant *S. aureus*. More work is needed to understand whether *S. pasteuri* can be used to facilitate enhancement of the microbiological safety of food and whether it can play a role in winemaking.

## 5. Conclusions

In all, 108 lactic acid bacterial strains were isolated from high-ethanol (around 17% (*v*/*v*)) Grenache wines; 16S rRNA and species-specific PCR showed that 104 of these were *O. oeni*, 3 were *L. hilgardii*, and 1 was an *S. pasteuri* isolate. An AFLP technique using *Hind*III and *Mse*I to digest the genomic DNA of *O. oeni* strains was developed for the first time to discriminate *O. oeni* strains. From the AFLP analysis and the growth of isolates, nine *O. oeni* strains and the three *L. hilgardii* strains were chosen for an assay of ethanol tolerance. Some *O. oeni* strains (G63, G46, G71, G39) survived and grew well in MRS-AJ with up to 17% (*v*/*v*) ethanol, whereas the commercial high-ethanol-tolerant reference strain did not grow. Further, *O. oeni* G63 could survive and grow for more than 168 h after inoculation into a 19% (*v*/*v*) ethanol medium. These results indicated that *O. oeni* G63, G46, G71, and G39 could have potential as MLF starter cultures for high-ethanol wines. Interestingly, all three *L. hilgardii* strains isolated could survive and grow well in MRS-AJ with 19% (*v*/*v*) ethanol. The potential of this species as a next-generation MLF starter culture is worthy of investigation.

## Figures and Tables

**Table 1:** Bacteria isolated from Grenache wines.

Origin	Barrel	Alcohol Content (*v*/*v*)	pH	Malic Acid (g/L)	Isolates
Barossa	AU2	16.8	3.76	1.50	G1, G2, G3, G89
Barossa	AU4	16.8	3.76	1.63	G4, G5, G6, G7, G8, G9, G10, G11, G12, G13, G14, G15, G16, G17, G18, G19, G20, G21, G22, G23, G24, G25, G26, G27, G28, G29, G30, G31, G32, G33, G34, G35, G36, G37, G38, G39, G40, G41, G42, G43, G90, G91, G92, G93, G94, G95, G96, G97, G100, G101, G102, G103
Barossa	AU6	16.9	3.71	1.50	G84, G85, G86, G87, G88, G98, G99, G104, G105
Barossa	AU12	17.0	3. 78	1.56	G44, G45, G46, G47, G48, G49, G50, G51, G52, G53, G54, G55, G56, G57, G58, G59, G60, G61, G62, G63, G64, G65, G66, G67, G68, G69, G70, G71, G72, G73, G74, G75, G76, G77, G78, G79, G80, G81, G82, G83, G106, G107, G108

**Table 2:** Identification of 108 isolates by 16S rRNA sequencing.

Identification	Number of Isolates	16S rRNA Sequencing Similarity (%)
*Oenococcus oeni*	104	99.42–100.00
*Lactobacillus hilgardii*	3	99.03–100.00
*Staphylococcus pasteuri*	1	99.46

**Table 3:** The average number of fragments obtained from eleven selective primer combinations and sequences to detect AFLPs among *O. oeni* strains isolated from Grenache wines. Bold type indicates the additional bases used for the selective primer.

Primer Pair	Sequences of Selective Primers	Fragment No.
MC-HT FAM	MC 5′-GATGAGTCCTGAGTAA**C**-3′ HT 5′-GACTGCGTACCAGCTT**T**-3′	57–81
MA-HT FAM	MA 5′-GATGAGTCCTGAGTAA**A**-3′ HT 5′-GACTGCGTACCAGCTT**T**-3′	76–85
MT-HT FAM	MT 5′-GATGAGTCCTGAGTAA**T**-3′ HT 5′-GACTGCGTACCAGCTT**T**-3′	70–85
MG-HT FAM	MG 5′-GATGAGTCCTGAGTAA**G**-3′ HT 5′-GACTGCGTACCAGCTT**T**-3′	57–64
MA-HC FAM	MA 5′-GATGAGTCCTGAGTAA**A**-3′ HC 5′-GACTGCGTACCAGCTT**C**-3′	54–62
MT-HC FAM	MT 5′-GATGAGTCCTGAGTAA**T**-3′ HC 5′-GACTGCGTACCAGCTT**C**-3′	52/74
MC-HC FAM	MC 5′-GATGAGTCCTGAGTAA**C**-3′ HC 5′-GACTGCGTACCAGCTT**C**-3′′	57–75
MG-HC FAM	MG 5′-GATGAGTCCTGAGTAA**G**-3′ HC 5′-GACTGCGTACCAGCTT**C**-3′	63–66
HG-MA HEX	HG 5′-GACTGCGTACCAGCTT**G**-3′ MA 5′-GATGAGTCCTGAGTAA**A**-3	52–78
HG-MT HEX	HG 5′-GACTGCGTACCAGCTT**G**-3′ MT 5′-GATGAGTCCTGAGTAA**T**-3	41–49
HA-MT HEX	HA 5′-GACTGCGTACCAGCTT**A**-3′ MT 5′-GATGAGTCCTGAGTAA**T**-3	34–49

**Table 4:** Reproducibility ratio of AFLP.

Primer	*O. oeni*			*L. hilgardii*		
	G13	G22	G63	G76	G102	G103
MA-HT FAM	99.60% ± 0.37	99.28% ± 0.19	99.71% ± 0.21	95.03% ± 0.37	96.17% ± 0.87	97.6% ± 0.93
MT-HT FAM	100%	99.70% ± 0.22	99.69% ± 0.40	95.65% ± 0.59	95.52% ± 0.61	96.58% ± 0.20

## Data Availability

Data is contained within the article or supplementary material.

## Supplementary Materials

The following supporting information can be downloaded at https://www.mdpi.com/article/10.3390/foods11091231/s1: Figure S1: Growth curve of Lactobacillus hilgardii strains in MRS-AJ contained19% ethanol (Valued by OD600)
