# Supplementary material for: Isolation and Characterization of High-Ethanol-Tolerance Lactic Acid Bacteria from Australian Wine
Source: Foods. 2022 Apr 25;11(9):1231. doi: 10.3390/foods11091231 (PMC9101528; doi:10.3390/foods11091231)
Supplement: Supplementary file 1 [file foods-11-01231-s001.zip › foods-1636853-SI.pdf]

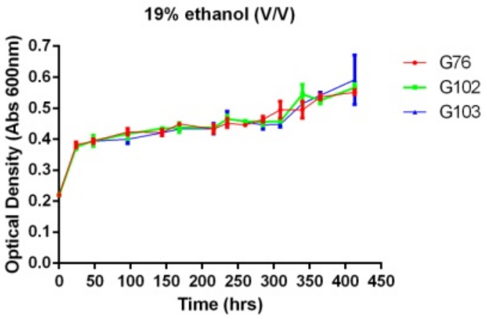

Figure S1. Growth curve of *Lactobacillus hilgardii* strains in MRS-AJ contained19% ethanol (Valued by OD_{600}).
